# Diagnosis of Oral Cancer With Deep Learning. A Comparative Test Accuracy Systematic Review

**DOI:** 10.1111/odi.15330

**Published:** 2025-03-31

**Authors:** Michele Nieri, Lapo Serni, Tommaso Clauser, Costanza Paoletti, Lorenzo Franchi

**Affiliations:** ^1^ Department of Experimental and Clinical Medicine University of Florence Italy; ^2^ Private Practice Florence Italy

**Keywords:** artificial intelligence, deep learning, oral cancer, oral medicine, oral pathology

## Abstract

**Objective:**

To directly compare the diagnostic accuracy of deep learning models with human experts and other diagnostic methods used for the clinical detection of oral cancer.

**Methods:**

Comparative diagnostic studies involving patients with photographic images of oral mucosal lesions (cancer or non‐cancer) were included. Only studies using deep learning methods were eligible. Medline, EMBASE, Scopus, Google Scholar, and ClinicalTrials.gov were searched until September 2024. QUADAS‐C assessed the risk of bias. A Bayesian meta‐analysis compared diagnostic test accuracy.

**Results:**

Eight studies were included, none of which had a low risk of bias. Three studies compared deep learning versus human experts. The difference in sensitivity favored deep learning by 0.024 (95% CI: −0.093, 0.206), while the difference in specificity favored human experts by −0.041 (95% CI: −0.218, 0.038). Two studies compared deep learning versus postgraduate medical students. The differences in sensitivity and specificity favored deep learning by 0.108 (95% CI: −0.038, 0.324) and by 0.010 (95% CI: −0.119, 0.111), respectively. Both comparisons provided low‐level evidence.

**Conclusions:**

Deep learning models showed comparable sensitivity and specificity to human experts. These models outperformed postgraduate medical students in terms of sensitivity. Prospective clinical trials are needed to evaluate the real‐world performance of deep learning models.

## 1 Introduction

Oral cancer still remains a significant global health concern. It represents the sixteenth malignancy in the world in terms of incidence and the fifteenth in terms of mortality, with growing trends among patients under 40 years of age (GCO; gco.iarc.fr/en). Although early detection is crucial for improving patient outcomes and reducing morbidity, 50% of cases are currently diagnosed at an advanced tumor stage. This diagnostic delay could be due to different reasons related to healthcare systems, patient characteristics, and intrinsic features of the disease (González‐Moles et al. 2022).

Automated detection of oral cancerous lesions using artificial intelligence (AI) offers a rapid, non‐invasive tool for immediate diagnostic results (Kim et al. 2022).

Machine learning (ML), a subset of AI, employs computational methods to learn patterns directly from data (Mahmood et al. 2020). Deep learning (DL), a specialized form of machine learning, enables algorithms to learn from input data without explicit programming (Mahmood et al. 2020). In DL, data (e.g., images) and their corresponding labels (e.g., cancer) are repeatedly fed into a neural network, and the model's parameters are adjusted to improve accuracy (Ahmed et al. 2021). DL has demonstrated remarkable capabilities, overtaking human performance in certain tasks, such as game playing (Silver et al. 2016).

Several systematic reviews (SRs) investigated the accuracy of AI and DL in predicting and diagnosing oral cancer, yielding promising results (Mahmood et al. 2020; Adeoye et al. 2021; Alabi et al. 2021; Khanagar et al. 2021; Kim et al. 2022; Di Fede et al. 2024; Li et al. 2024; Malhotra et al. 2024; Rokhshad et al. 2024; Sahoo et al. 2024; Warin and Suebnukarn 2024).

However, to our knowledge, previous SRs have not directly compared DL algorithms with other diagnostic methods, including human experts from various healthcare specialties and other ML architectures. Consequently, the potential of DL as an additional tool in oral cancer screening and diagnosis remains unclear (Bossuyt et al. 2006; Vali et al. 2021). Furthermore, various DL architectures, including ResNet, EfficientNet, DenseNet, and VGG, have been utilized for photograph‐based oral cancer diagnosis (Sharma et al. 2022). A direct comparison of their performance in diagnosing oral cancer would provide valuable insights.

The primary aim of this SR was to compare directly the diagnostic accuracy of DL models with that of human experts and other diagnostic methods. A secondary aim was to compare directly the diagnostic accuracy of different DL architectures.

## 2 Materials and Methods

### 2.1 Protocol and Registration

The SR and meta‐analysis protocol was prospectively registered on PROSPERO (ID code: CRD42023471929). This review was conducted in accordance with the Preferred Reporting Items for Systematic Reviews and Meta‐Analyses—Diagnostic Test Accuracy (PRISMA‐DTA) guidelines (Salameh et al. 2020).

### 2.2 Eligibility Criteria

The eligibility criteria were set as follows:

- P—Population: patients with photographic images of oral mucosal lesions and with a histological diagnosis of the oral lesions (cancer or non‐cancer). Any age, gender, or setting.
- I—Intervention: DL algorithms applied to clinical images of oral mucosal lesions for the initial diagnosis of oral cancer. This included all DL architectures and platforms employing such models.
- C—Comparison: different architectures of ML, including other DL algorithms or other diagnostic tests used for the diagnosis of oral cancer (e.g., clinical human evaluation, human evaluation of pictures of the lesions, other tools based on pictures, diagnostic screening tests such as toluidine blue staining test or chemiluminescent light test, performed ‘in vivo’ on oral lesions, etc.).
- O—Outcome: sensitivity and specificity of the DL and of the comparative diagnostic method. Difference in sensitivity and specificity between DL and the comparative diagnostic method. The reference standard for the tests was the histological diagnosis of the oral lesion. The cost of the diagnostic procedures was a secondary outcome.
- S—Study design: diagnostic and observational studies. Any language. Only direct comparisons between diagnostic methods were included. Studies considering Oral Potentially Malignant Disorders (OPMDs) were included only if they also analyzed oral cancer images.

### 2.3 Exclusion Criteria

The exclusion criteria were as follows:

- Non‐clinical studies or SRs;
- Animal studies;
- Studies reporting a single diagnostic intervention without a comparison.
- Studies not reporting the histological diagnosis of the oral lesions (e.g., studies using images collected from the internet without a confirmed histological evaluation).

### 2.4 Information Sources and Search

The literature search was conducted using Medline (via PubMed), EMBASE, and Scopus. Grey literature was also consulted through Google Scholar and ClinicalTrials.gov. The search period was from January 1, 2000 to September 5, 2024. The search strategies used for each database are listed in Table S1.

### 2.5 Study Selection

The results obtained from the databases were screened for duplicates using DOI matching on an Excel spreadsheet (Microsoft Corporation). Titles and abstracts were screened by two independent reviewers (LS and TC). When either one or both authors required a full‐text evaluation, the full text was screened for eligibility criteria. Studies meeting all the eligibility criteria, as determined by both reviewers, were included. Disagreements were resolved by a third reviewer (MN).

Two independent reviews (MN and LS) conducted a full‐text review of all eligible studies for final inclusion. Disagreements were resolved by a third reviewer (TC).

Cohen's kappa for titles/abstracts and full‐texts screening was calculated.

### 2.6 Data Collection Process

Two independent reviewers (MN and LS) completed a standardized form for each included study, recording the information listed below.

Study characteristics: reference, year of publication, first author's nationality, study setting, funding source, study design, number and type of diagnostic methods compared, type of oral lesions included, sample size for the learning and test phases.

Diagnostic performance metrics: sensitivity, specificity, true positive (TP), true negative (TN), false positive (FP), false negative (FN), area under the curve (AUC), cost of the diagnostic procedure.

When necessary, the corresponding authors of the included studies were contacted via email to obtain additional information.

### 2.7 Definition for Data Extraction and Diagnostic Accuracy Measures

True positive (TP): A diagnosis of oral cancer confirmed by histological examination.

True Negative (TN): A diagnosis of a non‐oral cancer confirmed by histological examination.

False Positive (FP): A diagnosis of oral cancer not confirmed by histological examination.

False Negative (FN): A diagnosis of a non‐oral cancer not confirmed by histological examination.

When TP, TN, FP, and FN were not directly reported, they were calculated using standard formulas based on sensitivity (or recall), specificity, disease prevalence, and total sample size. Corresponding authors were contacted via email when clarification was needed.

Comparative measures between two or more diagnostic tests were extracted, including differences in sensitivity and specificity, along with their corresponding confidence intervals and p‐values.

Differences between compared diagnostic tests were performed in terms of differences in sensitivity and differences in specificity.

### 2.8 Risk of Bias and Applicability

Risk of bias was evaluated independently by two reviewers (MN and LS). Disagreements were resolved by a third reviewer (TC).

QUADAS‐C was used for risk of bias assessment (Yang et al. 2021a). QUADAS‐C is an extension of QUADAS‐2 (Whiting et al. 2011) specifically designed for evaluating the risk of bias in comparative diagnostic test accuracy studies. QUADAS‐C retains the same four‐domain structure of QUADAS‐2 (‘Patient Selection’, ‘Index Test’, ‘Reference Standard’, and ‘Flow and Timing’) and is comprised of additional questions to each QUADAS‐2 domain (Yang et al. 2021a). Each domain can be judged as ‘low’, ‘high’, or ‘unclear’ risk of bias. An ‘overall’ risk of bias judgment was assigned based on the assessment of all domains: low overall risk of bias if all domains were at low risk of bias, and high or unclear overall risk of bias if one or more domains were at high or unclear risk of bias, respectively.

The GRADE approach for comparative test accuracy was used to assess the quality of evidence for each comparison (Yang et al. 2021b).

### 2.9 Synthesis of Results and Meta‐Analysis

A meta‐analysis was conducted for comparison with at least two studies. Different DL architectures were grouped when compared to non‐DL architecture (e.g., human experts).

A Bayesian meta‐analysis software (Metabayes DTA) for comparative accuracy of diagnostic tests was used (Cerullo et al. 2023). Raw data (TP, TN, FP, FN) were extracted from each study. The default uninformative prior distributions were used. For logit sensitivities and specificities—normal distribution with mean 0 and SD of 1, equivalent to a 95% prior interval of (0.05, 0.95) on the probability scale. For between‐study SD's of logit sensitivities and specificities—truncated (at 0) normal distribution with mean 0 and SD of 1, equivalent to a 95% prior interval of (0.03, 2.25).

For between‐study correlation between study‐specific logit sensitivities and specificities—LKJ(2) prior, equivalent to 95% prior interval of (−0.8, 0.8). A bivariate approach was applied.

Summary receiver operating characteristic (sROC) plot and vertical bars with the 95% credible intervals were plotted for each comparison. Using a bivariate Bayesian approach, posterior medians and 95% credible intervals were calculated for sensitivity, specificity, and the differences between them. A difference was considered statistically non‐significant if its 95% credible interval included zero.

### 2.10 Additional Analyses

Subgroup additional meta‐analysis was planned only for low risk of bias studies.

A robustness analysis was conducted to assess the impact of the uninformative prior assumption. Alternative prior distributions were examined for logit sensitivities and specificities with a normal distribution with a mean 1.39 (corresponding to 80% sensitivity and specificity) and a standard deviation of 2. A separate robustness analysis was performed using a conventional frequentist meta‐analysis approach. This analysis employed the Mada package in R, utilizing a bivariate diagnostic random‐effects meta‐analysis with the REML estimation method.

## 3 Results

### 3.1 Study Selection

**FIGURE 1:**
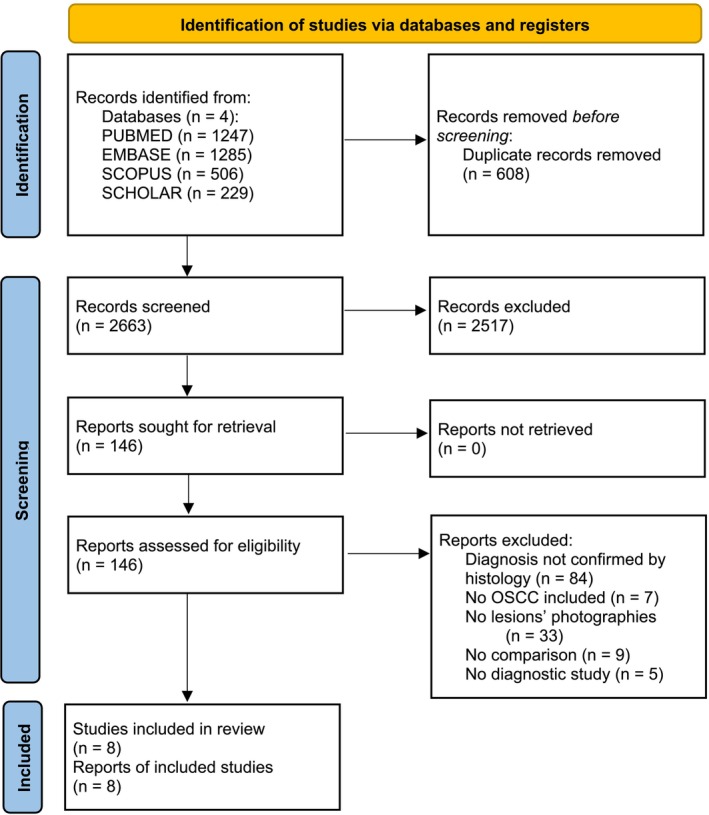
PRISMA 2020 flow diagram. Flow diagram showing the identification and selection process of the included studies.

The initial search identified 3271 studies and, after duplicates removal, 2663 studies were screened by title and abstract. Of these, 2517 studies were excluded, while 146 were assessed by full‐text review. Cohen's kappa for titles/abstracts screening was 0.96, 95% CI from 0.93 to 0.99. At the end of the selection process, 84 studies were excluded due to a lack of histological confirmation of the lesions depicted in the clinical images, 7 were excluded as non‐OSCC studies, 33 because they did not consider clinical photographic images of oral cancer, 9 because they were non‐comparative studies, and 5 because they were not diagnostic study (e.g., letter to editor, review, etc.) Consequently, 8 studies were included in this SR and 3 in the meta‐analysis (Figure 1). Cohen's kappa for full‐texts screening was 0.83, 95% CI from 0.64 to 0.99. There were no specific instances where the third reviewer was necessary to resolve disagreements between the two main reviewers.

### 3.2 Study Characteristics

Table 1 provides an overview of the characteristics of the included studies, all of which employed a retrospective study design. Four studies directly compared the performance of DL models to human evaluation (Fu et al. 2020; Warin et al. 2022; Dinesh et al. 2023; Ye et al. 2024). The remaining four studies focused on comparing the performance of different DL architectures (Jubair et al. 2022; Sharma et al. 2022; Mira et al. 2024; Rabinovici‐Cohen et al. 2024). Studies comparing DL with tissue autofluorescence, toluidine blue staining, or other diagnostic tools were not identified.

**TABLE 1:** Study characteristics.

Study	Country	Study design	Funding	Setting	Oral lesions	Index test	Comparators	Sample size
Fu et al. (2020)	China	Multicentric Retrospective	No funding	Public General and Stomatology Hospitals	Oral cancer lesions (OSCC, non‐OSCC malignancies, and dysplasia) vs. Others (benign lesions and normal mucosa)	DenseNet	7 Oral cancer specialists 7 Postgraduate medical students 7 Undergraduates non‐medical students	666
Jubair et al. (2022)	Jordan	One center Retrospective	Public	Not specified	Suspicious lesions of the tongue (OSCC and dysplasia) vs. Benign lesions of the tongue	EfficientNet‐BO	VGG‐19 ResNet‐101	100
Sharma et al. (2022)	India	One center Retrospective	Public	Public Department of Oral Medicine and Maxillofacial Surgery	OSCC vs. OPMD vs. Normal mucosa	VGG‐19	VGG16 MobileNet InceptionV3 ResNet50	25
Warin et al. (2022)	Thailand	Multicentric Retrospective	Public	Public Oral and Maxillofacial Surgical Centers	OSCC vs. OPMD vs. Non‐pathological oral images	DenseNet‐169 (cross‐validation)	ResNet‐101 (cross‐validation) SquizeNet (cross‐validation) Swin‐S (cross‐validation) 10 Board certified oral and maxillofacial surgeons 10 General practitioners	196 (cross‐validation)
Dinesh et al. (2023)	India	One center Retrospective	No external funding	Public Department of Oral Pathology	OSCC and OPMD vs. Non‐pathological oral images	Roboflow	2 Expert oral and maxillofacial specialists	60
Mira et al. (2024)	Egypt	One center Retrospective	No external funding	Public outpatient dental clinic	Oral cancer vs. High‐risk OPMD vs. Low‐risk OPMD vs. Aphthous ulcer vs. Normal mucosa	HRNet‐W18	VGG‐16 ResNet‐50 DenseNet‐169	455
Rabinovici‐Cohen et al. (2024)	Israel	One center Retrospective	No external funding	Public general hospital	OSCC vs. Mimic‐cancer lesions vs. Benign lesions/diseases vs. Normal mucosa	CNN (2D ResNet‐18)	OCNN (ResNet50) CNN Meta OCNN Meta CNN Meta + ISIC Pretrainer	366
Ye et al. (2024)	China	Multicentric Retrospective	Public	Public hospital	OSCC vs. Oral lichen planus vs. Oral leukoplakia vs. Normal oral mucosa	YOLOX	Senior group (2 Specialists and 1 general dentist with over 10 years of experience) Intermediate group (2 Specialists and 1 general dentist with under 10 years of experience) Student group (3 medical graduate students) Dental hospital group (5 specialists) General hospital group (6 general dentists) Community hospital group (4 general dentists)	120

Abbreviations: OPMD, oral potentially malignant disorders; OSCC, oral squamous cell carcinoma.

### 3.3 Risk of Bias and Applicability

The risk of bias and applicability concerns for each study were evaluated and are presented in Table 2. No study was judged at low risk of bias. Three studies were assessed as having an unclear risk of bias (Fu et al. 2020; Sharma et al. 2022; Jubair et al. 2022), whereas the remaining four studies were considered to be at high risk of bias. The main reasons for assessing studies as having a high or unclear risk of bias were ‘patient selection’ and ‘index test’. Many studies included images of healthy mucosa in the test dataset. One study (Sharma et al. 2022) tested different DL algorithms using different datasets. On the other hand, three studies (Warin et al. 2022; Mira et al. 2024; Rabinovici‐Cohen et al. 2024), tested various DL algorithms through a cross‐validation method, using images already present in the training and validation dataset.

**TABLE 2:** QUADAS‐C judgment for risk of bias of included studies.

Study	Risk of bias (QUADAS‐2)				Applicability concerns (QUADAS‐2)			Risk of bias (QUADAS‐C)				Overall risk of bias
	P	I	R	FT	P	I	R	P	I	R	FT	
Fu et al. (2020)	?	✓	✓	✓	✓	✓	✓	?	✓	✓	✓	?
Jubair et al. (2022)	?	✓	✓	✓	✓	✓	✓	?	✓	✓	✓	?
Sharma et al. (2022)	?	?	✓	✓	✓	✓	✓	?	?	✓	✓	?
Warin et al. (2022)	?	✗	✓	✓	✓	✗	✓	?	✗	✓	✓	✗
Dinesh et al. (2023)	✗	✓	✓	✓	✓	✓	✓	✗	✓	✓	✓	✗
Mira et al. (2024)	?	✓	✓	✗	✓	✓	✓	?	?	✓	✗	✗
Rabinovici‐Cohen et al. (2024)	✓	✗	✓	✓	✓	✓	✓	✓	✗	✓	✓	✗
Ye et al. (2024)	✗	✓	✓	?	✓	✓	✓	✗	✓	✓	?	✗

*Note:* ?, indicates unclear risk; ✓, indicates low risk; ✗, indicates high risk. Abbreviations: FT, flow and timing; I, index test; P, patient selection; R, reference standard.

### 3.4 Results of Individual Studies

Fu et al. (2020) compared a DL algorithm with 7 oral cancer specialists, 7 postgraduate medical students, and 7 undergraduate non‐medical students. Sensitivity and specificity with their 95% confidence intervals were reported in Table 3. The DL algorithm yielded comparable performance to that of human specialists and better performance than the average of postgraduate medical and non‐medical students in terms of sensitivity.

**TABLE 3:** Sensitivity and specificity of each comparison as reported in the original study.

Study	Test comparison	Sensitivity (95% CI)	Specificity (95% CI)
Fu et al. (2020)	DenseNet	91.0 (89.9; 94.1)	93.5 (90.9; 96.0)
	Oral cancer specialists	91.7 (89.8; 93.4)	93.1 (91.4; 94.8)
	Postgraduate medical students	83.1 (80.7; 85.4)	90.7 (88.9; 92.4)
	Undergraduates non‐medical students	76.6 (74.3; 78.8)	77.9 (75.9; 79.7)
Jubair et al. (2022)	EfficientNet‐BO	86.7 (80.4; 93.3)	84.5 (78.9; 91.5)
	VGG‐19	86.4 (77.6; 92.7)	81.5 (75.0; 88.1)
	ResNet‐101	83.9 (78.2; 92.7)	84.4 (77.0; 94.6)
Sharma et al. (2022)	VGG‐19	42.8^a^	88.8^a^
	VGG‐16	55.5	93.7
	MobileNet	83.3	84.2
	Inception‐V3	37.5	94.1
	ResNet‐50	0.0	100
Warin et al. (2022)	DenseNet‐169 (cross‐validation)	99	99
	ResNet‐101 (cross‐validation)	92	94
	SquizeNet (cross‐validation)	72	92
	Swin‐S (cross‐validation)	73	83
	Oral and maxillofacial surgeons	90	89
	General practitioners	77	87
Dinesh et al. (2023)	Roboflow	85^a^	80^a^
	Oral and maxillofacial specialists	90	100
Mira et al. (2024)	HRNet‐W18	90^a^	99^a^
	VGG‐16	—	—
	ResNet‐50	—	—
	DenseNet‐169	—	—
Rabinovici‐Cohen et al. (2024)	CNN (2D ResNet‐18)	—	—
	OCNN (ResNet50)	91	9
	CNN Meta	100	81
	OCNN Meta	—	—
	CNN Meta + ISIC Pretrained	—	—
Ye et al. (2024)	YOLOX	96.67 (93.45; 99.88)	91.11 (86.02; 96.20)
	Senior group	77.78 (70.35; 85.21)	97.41 (94.79; 100.00)
	Intermediate group	78.89 (71.59; 86.18)	95.56 (91.87; 99.24)
	Student group	72.22 (64.21; 80.23)	94.82 (91.91; 97.72)
	Dental hospital group	72.67 (66.33; 79.00)	92.22 (88.38; 96.06)
	General hospital group	53.89 (46.90; 60.88)	84.63 (78.86; 90.40)
	Community hospital group	57.50 (49.68; 65.32)	90.28 (85.19; 95.37)

^a^ Sensitivity and specificity were calculated from the confusion matrices.

Jubair et al. (2022) compared EfficientNet‐BO architecture with VGG‐19 and ResNet‐101 transfer learning models. Their performances were similar (Table 3).

Sharma et al. (2022) compared VGG‐19, VGG‐16, MobileNet, Inception‐V3, and ResNet‐50. In this study, a classification of three classes was considered as follows: malignant lesions, normal mucosa, and pre‐malignant lesions. For this reason, the authors reported only the accuracy that was 76% for VGG‐19, 72% for VGG‐16, 72% for MobileNet, 68% for Inception‐V3, and 36% for ResNet‐50. In Table 3, sensitivity and specificity were calculated from the confusion matrix considering malignant vs. normal and pre‐malignant lesions.

Warin et al. (2022) compared DenseNet‐169 (cross‐validation), ResNet‐101 (cross‐validation), SquizeNet (cross‐validation), Swin‐S (cross‐validation), board certified oral and maxillofacial surgeons, and general practitioners. Results were reported in Table 3. Several data were unclear in the original article, and the authors were contacted to clarify some aspects of the study. This study was excluded from the comparison between DL algorithms and humans because the performance of the DL algorithms was derived from cross‐validation and not tested on an external set of images. This study was considered, instead, in the comparison between different algorithms. DenseNet‐169 and ResNet‐101 outperformed SquizeNet and Swin‐S.

Dinesh et al. (2023) conducted a comparative diagnostic study between Roboflow software (Roboflow Inc., USA) and two expert oral and maxillofacial specialists. Ambiguities within the original report necessitated communication with the authors via email to obtain clarification on certain aspects of the study. Table 3 presents the sensitivity and specificity, calculated from the clarified confusion matrix provided by the authors.

Mira et al. (2024) compared HRNet‐W18, VGG‐16, ResNet‐50, and DenseNet‐169. Sensitivity and specificity were reported only for the multiclass performance of the different methods. The confusion matrix to calculate the sensitivity and specificity for oral cancer was reported only for the HRNet‐W18. It was not possible to contact the authors to obtain the confusion matrix of the other methods. HRNet‐W18 outperformed other models, except for specificity, similar to DenseNet‐169.

Rabinovici‐Cohen et al. (2024) compared CNN (2D ResNet‐18), OCNN (ResNet50), CNN Meta, OCNN Meta, and CNN Meta + ISIC Pretrained. The AUCs were, respectively, 0.87, 0.92, 0.84, 0.90, and 0.84. In this study, the authors computed in the validation set the threshold for sensitivity = 0.9 operation points and then used that threshold to calculate the sensitivity and specificity on the holdout test set. These metrics were reported for the CNN Meta and OCNN models and are presented in Table 3. The authors constructed a posteriori an ensemble method based on the CNN Meta and OCNN models. The results of this ensemble model were not reported in this SR because it was not compared a priori with the other methods.

Ye et al. (2024) compared the performance of YOLOX with various groups of humans categorized based on their level of experience and the type of hospital they were affiliated with. The groups included a senior group, an intermediate group, a student group, a dental hospital group, a general hospital group, and a community hospital group. Sensitivity and specificity results for each group are presented in Table 3.

Data regarding the cost of the diagnostic procedures were not identified in the selected studies.

### 3.5 Synthesis of Results

Meta‐analysis was conducted for two comparisons:

1. DL vs. Human Experts: three studies (Fu et al. 2020; Dinesh et al. 2023; Ye et al. 2024) were included.
2. DL vs. Postgraduate Medical Students: two studies (Fu et al. 2020; Ye et al. 2024) were included.

Meta‐analysis was not feasible for comparing different DL models due to a lack of studies directly comparing the same models.

### 3.6 Deep Learning Versus Human Experts

**FIGURE 2:**
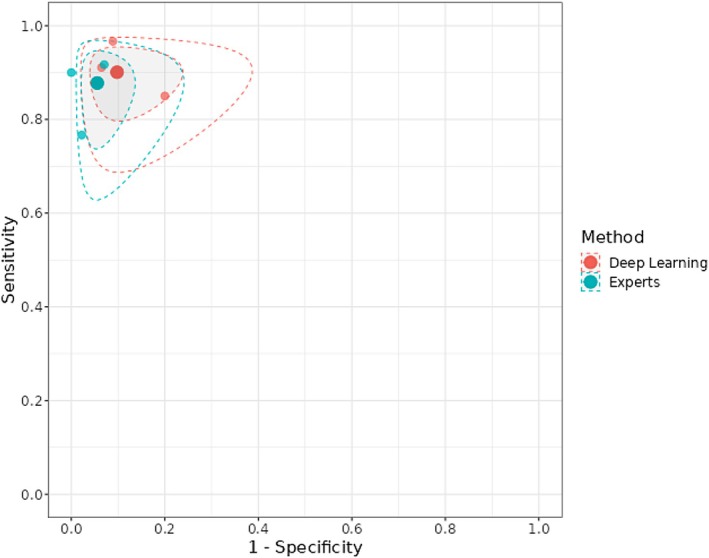
sROC plot for Deep Learning versus Experts. The solid big circles represent the summary sensitivity and specificity for each method. The dotted line(s) represent the 95% prediction region from the bivariate model; the grayed‐out area(s) represent the 95% credible region from the bivariate model.

Three studies (Fu et al. 2020; Dinesh et al. 2023; Ye et al. 2024) were included. The sROC plot is reported in Figure 2.

**FIGURE 3:**
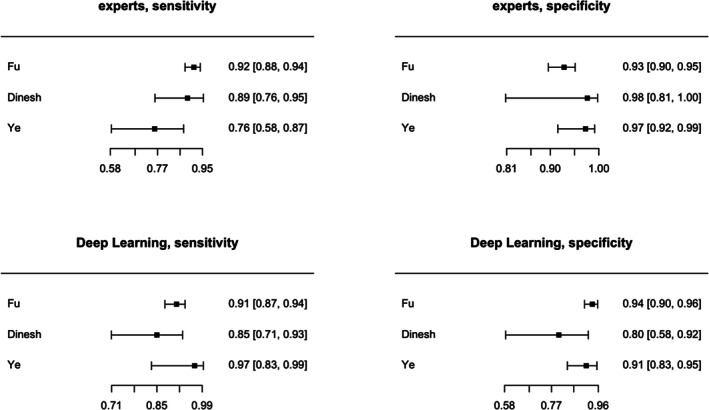
Forest plots of Deep Learning and experts.

Posterior median sensitivity was 0.901 (95% credible interval 0.767; 0.951) for DL and 0.878 (95% CI: 0.682; 0.935) for human experts (Figure 3, Figure S1). Posterior median for the difference in sensibility was 0.024 (95% CI: −0.093; 0.206) favoring DL.

Posterior median specificity was 0.902 (95% CI: 0.714; 0.948) for DL and 0.945 (95% CI: 0.852; 0.979) for human experts (Figure 3, Figure S1). Posterior median for difference in specificity was −0.041 (95% CI: −0.218; 0.038) favoring human experts.

### 3.7 Deep Learning Versus Postgraduate Medical Students

**FIGURE 4:**
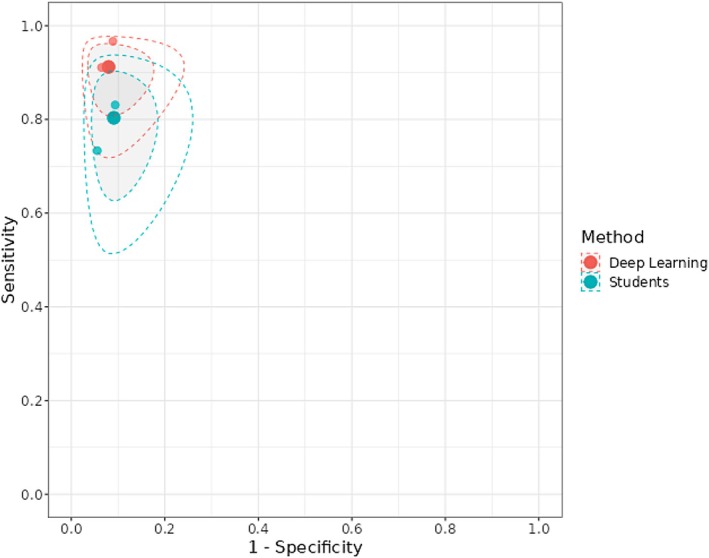
sROC plot for Deep Learning versus Postgraduate Medical Students. The solid big circles represent the summary sensitivity and specificity for each method. The dotted line(s) represent the 95% prediction region from the bivariate model; the grayed out area(s) represent the 95% credible region from the bivariate model.

Two studies (Fu et al. 2020; Ye et al. 2024) were included. The sROC plot is reported in Figure 4.

**FIGURE 5:**
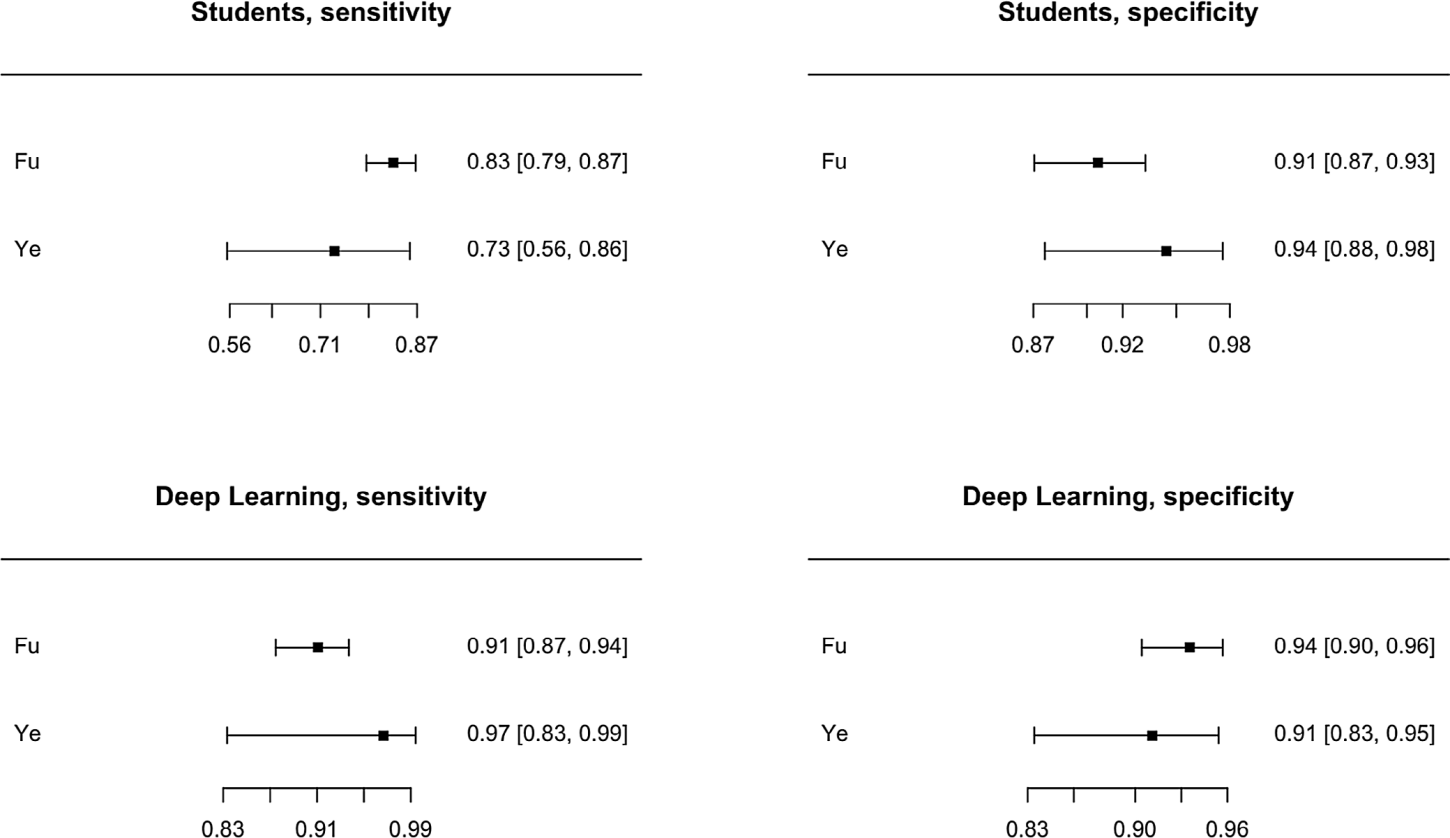
Forest plots of Deep Learning and students.

Posterior median sensitivity was 0.912 (95% CI: 0.760; 0.958) for DL and 0.803 (95% CI: 0.557; 0.893) for postgraduate medical students (Figure 5, Figure S2). Posterior median for the difference in sensibility was 0.108 (95% CI: −0.038; 0.324) favoring DL.

Posterior median specificity was 0.920 (0.755; 0.960) for DL and 0.909 (95% CI: 0.780; 0.951) for postgraduate medical students (Figure 5, Figure S2). Posterior median for the difference in specificity was 0.010 (95% CI: −0.119; 0.111) favoring DL.

### 3.8 GRADE Evaluation

GRADE evaluation was reported in Table 4. Both results of the comparison between DL and human expert or postgraduate medical students were rated at a low level of evidence.

**TABLE 4:** Grade profile for the comparison between deep learning and human experts and deep learning and postgraduate medical students.

Comparison	Study design	Risk of bias	Indirectness	Inconsistency	Imprecision	Publication bias	Outcome	DL	Ex	Diff	95% CI	Certainty
DL vs. Ex 3 studies	Fully paired	Serious	Serious	Not serious	Not serious	Undetected	True Positive	90	88	2 more	9 fewer; 21 more	++−− Low
							True Negatives	812	850	38 fewer	196 fewer; 34 more	

Comparison	Study design	Risk of bias	Indirectness	Inconsistency	Imprecision	Publication bias	Outcome	DL	St	Diff	95% CI	Certainty
DL vs. St 2 studies	Fully paired	Serious	Serious	Not serious	Not serious	Undetected	True Positive	91	80	11 more	4 fewer; 32 more	++−− Low
							True Negatives	828	818	10 morer	107 fewer; 100 more	

*Note:* True positives and true negatives were calculated considering 1000 lesions with a cancer prevalence of 10%. Abbreviations: 95% CI, 95% Credible Interval; Diff, Difference; DL, Deep Learning; Ex, Experts.

### 3.9 Additional Analysis

Subgroup analysis was not performed for low‐risk‐of‐bias studies because no study was assessed as having a low risk of bias.

The robustness analysis using the alternative prior distributions yielded similar results to the main analysis. For DL versus human experts, the difference in sensitivity was 0.021, and the difference in specificity was −0.040. For DL versus postgraduate medical students, the difference in sensitivity was 0.105, and the difference in specificity was 0.009.

The robustness analysis using the frequentist meta‐analysis package (Mada) also showed similar results to the Bayesian analysis. For DL versus human experts, the sensitivities were 0.895 and 0.870, respectively (difference: 0.025), and the specificities were 0.905 and 0.953, respectively (difference: −0.048). The AUCs were 0.943 for DL and 0.967 for human experts. For DL versus postgraduate medical students, the sensitivities were 0.928 and 0.798, respectively (difference: 0.130), and the specificities were 0.928 and 0.923, respectively (difference: 0.005). The AUCs were 0.961 for DL and 0.935 for postgraduate medical students.

## 4 Discussion

Diagnostic delay of oral cancer still represents a critical issue. At the moment, none of the adjunctive diagnostic screening tests, such as vital staining, oral cytology, light‐based detection, and oral spectroscopy could be considered sufficiently reliable (Walsh et al. 2021). In this context, DL represents a recent innovation.

This SR investigated the diagnostic accuracy of DL models for oral cancer, comparing them to human experts and other diagnostic methods. None of the included studies were considered to be at low risk of bias, raising concerns about the generalizability of the findings.

The review identified a limited number of studies directly comparing DL models with human experts for diagnosing oral cancer. A meta‐analysis suggested that DL algorithms might have comparable sensitivity (ability to detect cancer) to human experts, but potentially lower specificity (ability to correctly identify healthy tissue or no cancer lesions).

DL models appeared to outperform postgraduate medical students in terms of sensitivity.

However, studies comparing different DL architectures were inconclusive due to the heterogeneity of the methods used.

Recently, several SRs were published on the diagnostic performance of AI or DL in detecting oral cancer using oral mucosal lesions on photographs (Di Fede et al. 2024; Li et al. 2024; Malhotra et al. 2024; Rokhshad et al. 2024; Sahoo et al. 2024; Warin and Suebnukarn 2024).

In one of these SRs (Di Fede et al. 2024) fourteen studies utilizing various DL algorithms for the detection and classification of oral lesions from clinical images were selected. Among these, three were included in the meta‐analysis. The estimated pooled sensitivity and specificity were 0.86 and 0.67, respectively.

Unlike the present SR, none of the previously published reviews on this topic were comparative test accuracy SRs focusing on direct comparisons between different diagnostic methods. Although between‐study comparisons (indirect comparisons) of test accuracy across studies, each evaluating a single index test, may provide some evidence of relative test performance, they are often limited by factors such as differences in study populations and methodologies (Yang et al. 2020). Such comparisons typically lead to lower certainty of the evidence (Yang et al. 2021b). As the development of recommendations requires consideration of alternative diagnostic methods, studies directly comparing the performance of two or more tests are generally more informative (Yang et al. 2021b). For this reason, we limited our review to diagnostic comparative studies.

In this review, DL methods demonstrated comparable sensitivity and specificity to human experts. This is a significant achievement for DL technology. However, it is important to note that all included studies were retrospective, relying on clinicians' diagnostic judgments based solely on images of oral lesions. This approach differs from real‐world clinical practice, where clinicians can incorporate additional factors such as patient age, sex, habits, and the physical characteristics of lesions (e.g., consistency, mobility, tenderness) to enhance diagnostic accuracy beyond what is achievable through image analysis alone. Consequently, prospective comparative studies involving real patients are crucial to fully evaluate the clinical utility of DL models in oral cancer diagnosis.

While DL models showed superior sensitivity compared to postgraduate medical students, their specificity was comparable. This is a notable achievement for DL models. Nonetheless, as with experts, prospective studies are needed to assess more accurately the comparative performance of these two methodologies.

Numerous DL architectures were evaluated across the included studies. However, a meta‐analysis was precluded by the lack of studies directly comparing identical models. The heterogeneity of methodologies further hampered definitive conclusions regarding the superiority of any single DL architecture. While some algorithms, such as DenseNet and YOLOX, showed promising sensitivity and specificity (Fu et al. 2020; Warin et al. 2022; Ye et al. 2024), the optimal architecture remains to be determined. External validation on novel and robust image datasets would allow a more comprehensive comparison of existing algorithms.

A limitation of this SR is that none of the included studies were considered to be at low risk of bias. Furthermore, all studies were retrospective, and diverse DL algorithms were aggregated for comparisons of DL versus human experts and DL versus postgraduate medical students. Moreover, two studies included in the meta‐analysis (Fu et al. 2020; Dinesh et al. 2023) provided sensitivities and specificities for the classification of ‘suspected lesions’ versus controls, grouping together both oral cancer and OPMDs in the same category, not considering them as separate entities as done by Ye et al. (2024). This may limit the generalizability of the results.

The potential cost‐effectiveness of DL compared to traditional diagnostic methods was not explored in this review, as the included studies did not investigate this aspect. This represents an important area for future research.

This discussion highlights the critical importance of dataset characteristics for the successful development and implementation of DL algorithms in oral cancer diagnosis. As previously emphasized, the following key features are essential for building robust, reliable, and clinically relevant DL systems:

- Lesions heterogeneity: Training datasets must include a broad spectrum of oral lesion types, both benign and malignant ones, to enable DL models to effectively distinguish different pathological entities. This “all‐encompassing” approach is crucial for developing generalizable diagnostic tools. Furthermore, DL models should be able to sub‐classify different OPMD lesions and to correctly diagnose common benign conditions such as geographic tongue and oral infections.
- Dataset Size and Multicentricity: Large, multicentric datasets are necessary to adequately train DL models and prevent overfitting. Collaborative efforts between research centers and hospitals are fundamental to building datasets of sufficient size and diversity.
- Histological Ground Truth: The inclusion of images with confirmed histological diagnoses (“gold standard”) is essential to guarantee the scientific reliability of DL models. Only through this approach will the algorithms be able to recognize pathological patterns with high accuracy.
- Integration of Clinical Data: As noted, incorporating clinical data (patient history, symptoms, lesion location and consistency, lymph node involvement) is crucial for creating comprehensive AI systems. This contextual information enhances diagnostic precision and allows for more personalized assessments.
- Specific Diagnoses and Specialistic Support: The ultimate goal is to develop algorithms capable of providing specific diagnoses, thus improving both the screening process performed by general practitioners and the final diagnosis assessed by specialists. This will improve diagnostic accuracy and streamline patient care pathways.
- Monitoring Lesion Evolution: The ability to track lesion progression over time, particularly for OPMDs such as leukoplakia, oral lichen planus, and proliferative verrucous leukoplakia, is another valuable feature. By comparing serial follow‐up images of the same lesion, DL algorithms can assist specialists in identifying malignant changes, supporting early diagnosis of oral cancer and timely therapeutic interventions.
- Prognosis and Treatment Strategies: Hopefully, future DL systems should not only provide diagnoses but also prognosis (predicting the risk of progression or malignant transformation) also suggest personalized treatment strategies. This predictive and prescriptive approach represents a significant advancement in precision medicine.

In conclusion, this SR highlights the potential of DL for oral cancer diagnosis. However, further high‐quality studies are required to validate its efficacy and to establish its role in comparison to existing diagnostic methods.

## Author Contributions

**Michele Nieri:** conceptualization, writing – original draft, methodology, data curation, formal analysis. **Lapo Serni:** writing – review and editing, data curation, conceptualization, investigation. **Tommaso Clauser:** investigation, data curation, supervision. **Costanza Paoletti:** investigation, supervision. **Lorenzo Franchi:** funding acquisition, supervision, project administration, writing – review and editing.

## Conflicts of Interest

The authors declare no conflicts of interest.

## Supporting information


Figure S1.

Figure S2.

Table S1.

## Data Availability

The data that support the findings of this study are available from the corresponding author upon reasonable request.
