# Supplementary figures and images for: Diagnosis of Oral Cancer With Deep Learning. A Comparative Test Accuracy Systematic Review
Source: Oral Dis. 2025 Mar 31;31(8):2368–81. doi: 10.1111/odi.15330 (PMC12423475; doi:10.1111/odi.15330)

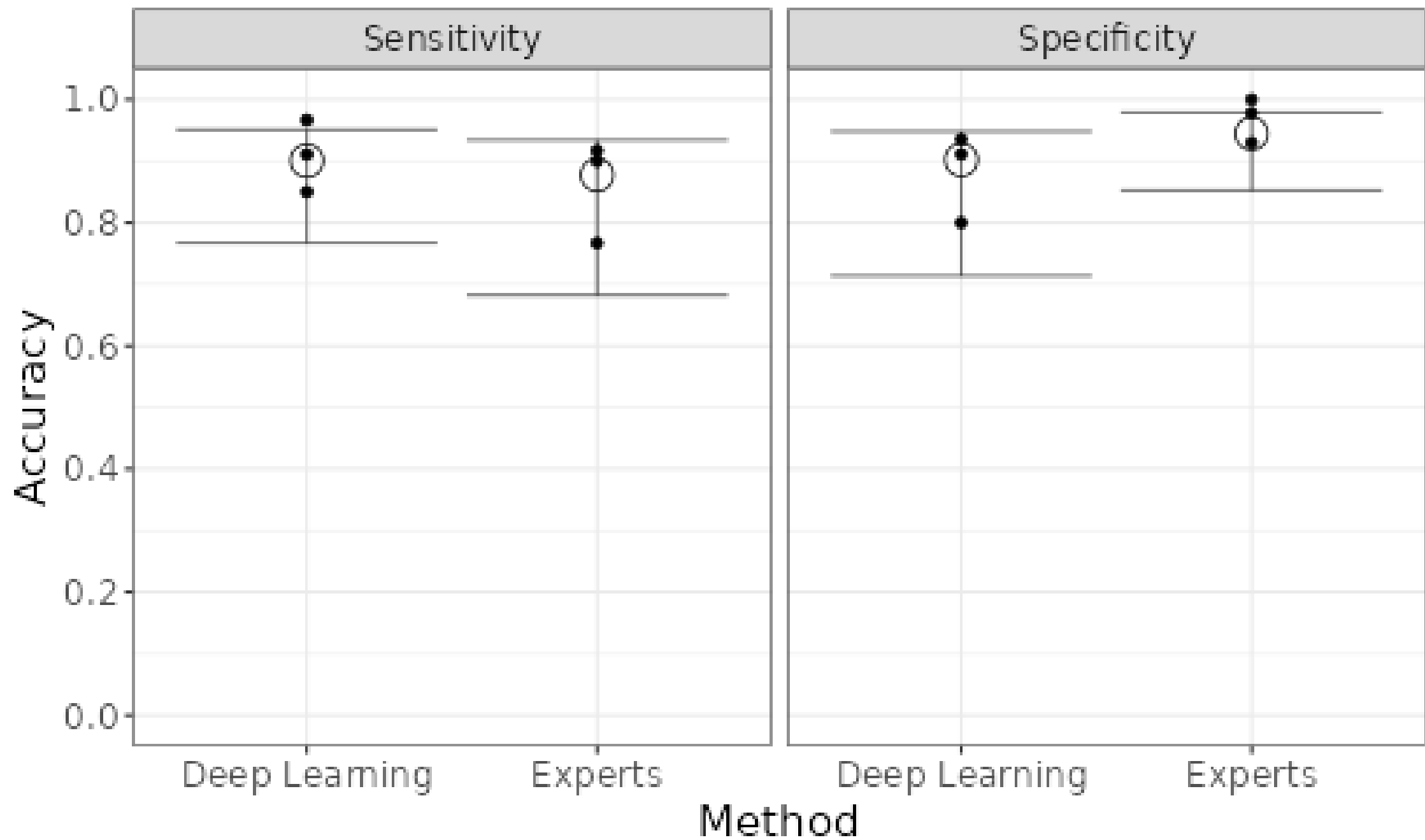

Supplement: Supplementary file 1 — Figure S1. [file ODI-31-2368-s001.pdf]

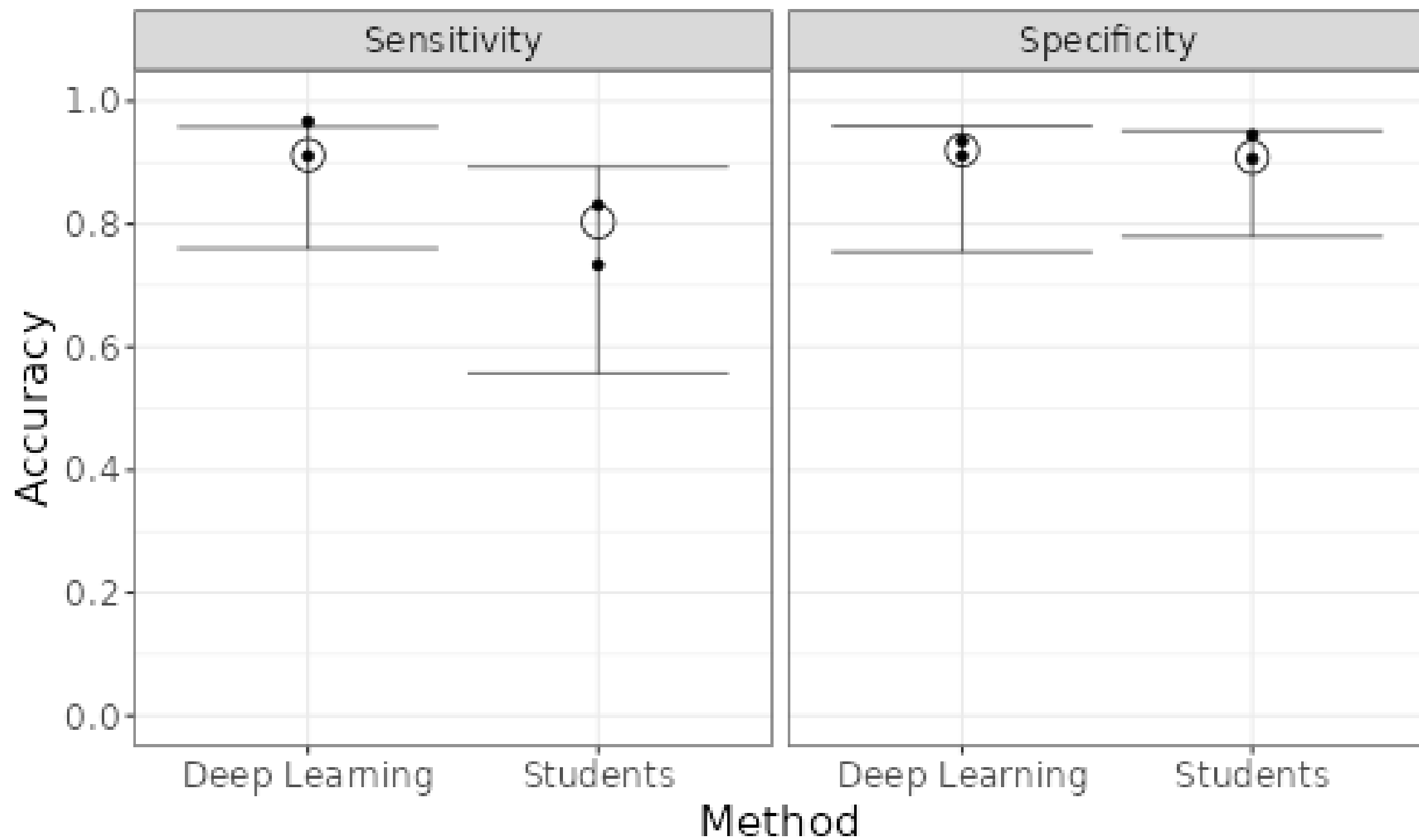

Supplement: Supplementary file 2 — Figure S2. [file ODI-31-2368-s003.pdf]
