# Supplementary material for: Diagnosis of Oral Cancer With Deep Learning. A Comparative Test Accuracy Systematic Review
Source: Oral Dis. 2025 Mar 31;31(8):2368–81. doi: 10.1111/odi.15330 (PMC12423475; doi:10.1111/odi.15330)
Supplement: Supplementary file 3 — Table S1. [file ODI-31-2368-s002.docx]

| Source | Search strategy |
| --- | --- |
| PubMed | (oral cancer OR oral carcinoma OR oral squamous cell carcinoma OR oral potentially malignant disorders OR oral tumor) AND (diagnosis OR detection) AND (deep learning OR artificial intelligence OR machine learning) |
| Embase | (oral AND cancer) OR (oral AND carcinoma) OR (oral AND squamous AND cell AND carcinoma) OR (oral AND potentially AND malignant AND disorders) OR (oral AND tumor) AND (diagnosis OR detection) AND ((deep AND learning) OR (artificial AND intelligence) OR (machine AND learning)) |
| Scopus | (oral AND cancer) OR (oral AND carcinoma) OR (oral AND squamous AND cell AND carcinoma) OR (oral AND potentially AND malignant AND disorders) OR (oral AND tumor) AND (diagnosis OR detection) AND ((deep AND learning) OR (artificial AND intelligence) OR (machine AND learning)) |
| Google Scholar | oral cancer deep learning. The search was conducted by two reviewers (CP and MN) independently and was halted after examining five consecutive pages that did not contain any additional pertinent article (Haddaway et al., 2015) |
| Clinicaltrials.gov | disease: oral cancer; other terms: diagnosis, deep learning |
